# Down-regulation of *p*-coumaroyl quinate/shikimate 3′-hydroxylase (C3′H) and cinnamate 4-hydroxylase (C4H) genes in the lignin biosynthetic pathway of *Eucalyptus urophylla* × *E. grandis* leads to improved sugar release

**DOI:** 10.1186/s13068-015-0316-x

**Published:** 2015-08-27

**Authors:** Robert W. Sykes, Erica L. Gjersing, Kirk Foutz, William H. Rottmann, Sean A. Kuhn, Cliff E. Foster, Angela Ziebell, Geoffrey B. Turner, Stephen R. Decker, Maud A. W. Hinchee, Mark F. Davis

**Affiliations:** National Bioenergy Center, National Renewable Energy Laboratory, 15013 Denver West Parkway, Golden, CO 80401-3393 USA; Biosciences Center, National Renewable Energy Laboratory, 15013 Denver West Parkway, Golden, CO 80401-3393 USA; ArborGen Inc., 2011 Broadbank Ct., Ridgeville, SC 29472 USA; Great Lakes Bioenergy Research Center, Michigan State University, East Lansing, MI 48824 USA

**Keywords:** *Eucalyptus urophylla* × *E. grandis*, Recalcitrance, Genetic modification, Lignin biosynthesis, Pretreatment

## Abstract

**Background:**

Lignocellulosic materials provide an attractive replacement for food-based crops used to produce ethanol. Understanding the interactions within the cell wall is vital to overcome the highly recalcitrant nature of biomass. One factor imparting plant cell wall recalcitrance is lignin, which can be manipulated by making changes in the lignin biosynthetic pathway. In this study, eucalyptus down-regulated in expression of cinnamate 4-hydroxylase (C4H, EC 1.14.13.11) or *p*-coumaroyl quinate/shikimate 3′-hydroxylase (C3′H, EC 1.14.13.36) were evaluated for cell wall composition and reduced recalcitrance.

**Results:**

Eucalyptus trees with down-regulated *C4H* or *C3′H* expression displayed lowered overall lignin content. The control samples had an average of 29.6 %, the *C3′H* reduced lines had an average of 21.7 %, and the *C4H* reduced lines had an average of 18.9 % lignin from wet chemical analysis. The *C3′H* and *C4H* down-regulated lines had different lignin compositions with average S/G/H ratios of 48.5/33.2/18.3 for the *C3′H* reduced lines and 59.0/39.8/1.2 for the *C4H* reduced lines, compared to the control with 65.9/33.2/1.0. Both the *C4H* and *C3′H* down-regulated lines had reduced recalcitrance as indicated by increased sugar release as determined using enzymatic conversion assays utilizing both no pretreatment and a hot water pretreatment.

**Conclusions:**

Lowering lignin content rather than altering sinapyl alcohol/coniferyl alcohol/4-coumaryl alcohol ratios was found to have the largest impact on reducing recalcitrance of the transgenic eucalyptus variants. The development of lower recalcitrance trees opens up the possibility of using alternative pretreatment strategies in biomass conversion processes that can reduce processing costs.

## Background

Commercial feedstocks for conversion to ethanol have traditionally been limited to corn starch and sugarcane, but the use of food crop feedstocks has generated concern due to their important role in the world food supply [1]. Cellulosic ethanol, produced from trees, biomass residues, and herbaceous and other non-food-source biomass is a sustainable alternative that does not use feedstocks that would otherwise compete with existing food supplies. However, using lignocellulosic feedstocks is hampered by the higher recalcitrance to ethanol bioconversion caused by the inaccessibility of the carbohydrate substrate due to the complex nature of the plant cell wall [2, 3] that contributes to its natural ability to resist decomposition by enzymes [2, 4, 5]. Transgenic plants with modified cell walls may provide a source of alternative lignocellulosic feedstock. Developing chemical processes to overcome recalcitrance and improve ethanol yields from lignocellulosic materials by improving conversion efficiencies is an ongoing area of research [6]. Recently, understanding the recalcitrant nature of biomass by exploring genetically modified feedstocks that can be more easily converted to biofuels has been explored [7–11].

**Fig. 1:**
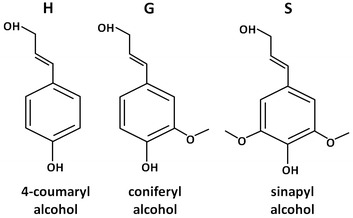
Chemical structures of starting alcohols used in the formation of lignin

One of the major contributors to biomass recalcitrance is believed to be the lignin present within the cell wall [2]. Lignin is essential to the plant as it provides the structure to cell walls and provides a barrier to natural plant pathogens. Lignin is formed by the polymerization of a combination of sinapyl alcohol (S), coniferyl alcohol (G), and 4-coumaryl alcohol (H) subunits (Fig. 1) [12]. The relative levels of S, G, and H lignin subunits are expressed as the S**/**G/H ratio. However, since H subunits are not found in significant amounts in woody species, the ratio of lignin subunits is typically expressed as the S/G ratio. H lignin is only present in very low levels in most plants, such as alfalfa (*Medicavo sativa*) = 4.9 % [7], Norway spruce (*Picea abies*) = 0.8 % [13], and hybrid poplar (*Populus alba* × *P. grandidentata*) = 0.2 % [14]. Lignin content, structure, and the ratio of the lignin precursors may impact the ability of enzymes to access the carbohydrates contained in the cell wall in biochemical processing using pretreatment to increase accessibility [3, 7, 10, 15]. Understanding the role of lignin content, structure, and molecular weight can identify ways to reduce the recalcitrant nature of biomass, minimize or eliminate pretreatment, and facilitate the development of cost-effective biochemical conversion processes for producing biofuels.

Plantation-grown trees, especially eucalyptus grown in Brazil, are already bioprocessed to produce chemical cellulose and pulp. Eucalyptus is considered the most productive tree for pulpwood production due to its biomass production rate and the properties of its wood. Improving the genetics of eucalyptus to improve pulping efficiency is a goal of the forest products industry, including traditional breeding [16], marker-assisted breeding [17], and genetically modified trees to reduce lignin content [18] and alter lignin monomer composition [19]. Advances in biotechnology have lowered the costs of gene mutation and genetic modification in trees. This has allowed tree improvement researchers to conduct functional screening of large numbers of transgenic trees a year in order to identify genetic approaches that alter cell wall composition and/or biopolymer structure, some leading to plants with lower recalcitrance. Reducing the lignin content of trees can result in lower chemical bleaching costs during the pulp and paper processing, which has provided a major impetus for exploring natural populations to identify trees with mutations in the lignin biosynthetic pathway. The lignin biosynthetic pathway has been extensively studied and there are a variety of avenues to explore when attempting to reduce plant cell wall recalcitrance [20]. Caffeic acid 3-*O*-methyltransferase (COMT) down-regulation has led to increased saccharification in sorghum (*Sorghum bicolor* (L.) Moench) [21], switchgrass (*Panicum virgatum*) [8], and alfalfa (*M. sativa*) [7]. Reduced recalcitrance lines have also demonstrated increased ethanol production in switchgrass lines in which *COMT* expression has been altered [22] and in poplar with down-regulated *p*-coumarate 3-hydroxylase or over-expressed ferulate 5-hydroxylase [10].

Cinnamate 4-hydroxylase (C4H, EC 1.14.13.11) and *p*-coumaroyl quinate/shikimate 3′-hydroxylase (C3′H, EC 1.14.13.36) are involved near the beginning of the lignin biosynthetic pathway and their down-regulation is, therefore, expected to reduce overall lignin content and potentially impact the S/G ratio [23, 24]. Many prior publications have referred to the latter enzyme as *p*-coumarate 3-hydroxylase; in this paper, C3′H is being used to mean either possibility. In alfalfa, down-regulation of the *C3′H* gene decreased lignin content, increased H lignin monomers, and decreased molecular weight, but caused little change in the S/G ratio [25, 26]. In a second alfalfa study, *C4H* down-regulated plants not only displayed lower lignin values, but also demonstrated reduced S/G ratios [27]. One notable difference between *C3′H* and *C4H* down-regulation in alfalfa occurs in the H lignin content, where plants with altered *C3′H* expression produced a large increase in H lignin, but plants with altered *C4H* expression displayed no significant change from the control. In poplar plants down-regulated in *C3′H* expression, total lignin decreased and H lignin increased while S/G ratios increased [14]. Down-regulation of *C4H* in tobacco (*Nicotiana tabacum*) displayed decreases in total lignin and slight decreases in S/G [28]. This paper deals with transgenic lines of hybrid eucalyptus (*Eucalyptus urophylla* × *E. grandis*) exhibiting down-regulated *C4H* or *C3′H* expression. Specifically, the trees were transformed using RNA interference (RNAi) constructs directed against *EgrC4H1*, the gene encoding the most strongly xylem-expressed C4H, or *EgrC3′H3*, the gene encoding the most strongly xylem-expressed C3′H [29].

## Results and discussion

### Selection of lines

After generating and field testing multiple lines of eucalyptus transformed using plasmids designed to reduce lignin content, wood from the trees was characterized by pyrolysis molecular beam mass spectrometry (PyMBMS) to identify lines with moderate levels of lignin reduction (approximately 30 % reduction). A subset of selected lines was established in a greenhouse, grown for 6 months, and characterized using quantitative polymerase chain reaction (qPCR) to verify that the target genes were indeed reduced in expression (Table 1). Four lines transformed using the pARB670 plasmid (*EgrC4H1* down-regulation) had an average *EgrC4H1* RNA expression level of 9.5 ± 1.0 % of that found in control samples, with a mild reduction of *EgrC3′H3* expression to 75 ± 6 % of control levels. Accordingly, three lines transformed using the pARB669 plasmid (*EgrC3′H3* down-regulation) had an average *EgrC3′H3* RNA expression level of 10.8 ± 3.6 % of that found in control samples, with a nonsignificant reduction of *EgrC4H1* expression. Although it was not possible to perform qPCR analysis on all lines due to losses of plants in tissue culture storage, these results suggest that the initial PyMBMS characterization allowed accurate selection of lines with the target genes reduced to similar levels.

**Table 1:** RT-PCR quantification of gene expression

Sample	Relative expression of C4H (Eucgr.H01844)	Relative expression of C3H (Eucgr.A02190)
Control 014	0.95	0.85
Control 018	0.98	1.08
Control 023	1.06	1.03
Control avg	1.00 ± 0.06	0.99 ± 0.12
C3′H 105	0.79	0.15
C3′H 113	0.68	0.093
C3′H 114	0.79	0.082
C3′H avg	0.75 ± 0.06*	0.108 ± 0.036*
C4H 091	0.11	0.76
C4H 092	0.092	1.22
C4H 093	0.091	0.69
C4H 098	0.087	0.62
C4H avg	0.095 ± 0.010*	0.82 ± 0.27

* Indicates significant difference from the control average at the *p* value <0.05 level

### Extractives content

Eucalyptus trees can have high levels of natural oils in the wood that could impact the methods used to determine sugar release. To remove these natural oils, a water and ethanol extraction was performed. After extraction, there was a reduction in the error associated with the sugar-release results. Average standard deviation for the unextracted wood was ±0.025 g/g, compared to ±0.011 g/g biomass for the extractives-free wood. Sugar-release results from the extractives-free wood were comparable to the unextracted wood with a R^2^ = 0.91 (data not shown).

### Starch removal

Starch content can artificially inflate sugar release results, falsely indicating lower recalcitrant samples [30]. To prevent starch content from effecting sugar release results, it can be removed. However, the starch content for all samples used in this study was determined to be lower than 1.2 %, with a measurement error of 1 %. Due to the low starch values measured, samples were run without a starch removal treatment.

### Cell wall composition

The results of the cell wall compositional analysis are shown in Table 2. The down-regulation of the *C3′H* and *C4H* genes in the lignin pathway is expected to result in a reduction of the lignin precursors necessary for normal lignin biosynthesis, potentially lowering the lignin content as well as altering the S/G/H ratio. Both the *C3′H* and *C4H* RNAi lines were observed to have significantly reduced lignin content compared to the untransformed control (Table 2). The *C3′H* down-regulated plants resulted in an average 26.7 % reduction of lignin content, while the *C4H* down-regulated plants had an average 36.1 % lignin content reduction. The reduction in lignin content for *C3′H* and *C4H* down-regulated lines was consistent with the findings of Chen and Dixon [7] in alfalfa where similar genetic transformations displayed a 37 % reduction for *C3′H* antisense lines and a 29 % reduction for *C4H* antisense lines. In eucalyptus, the reduction in lignin content in both *C3′H* and *C4H* down-regulated lines was offset by increases in the measured extractives, xylose, and acetyl contents (Table 2). Average glucose content of the *C4H* down-regulated lines (43.0 %) was increased compared to the control (40.2 %), while the glucose content for the *C3′H* down-regulated lines was relatively unchanged (39.7 %) (Table 2). Galactose contents for both *C3′H* and *C4H* down-regulated lines remained relatively unchanged compared to the control. The increase in xylose and acetyl content for both *C3′H* and *C4H* RNAi lines is consistent with *O*-acetyl-(4-*O*-methylglucurono)xylan, the primary hardwood hemicellulose, comprising a larger fraction of the cell wall. There was a decrease in arabinose content for the *C4H* down-regulated lines, although the arabinose levels measured were close to the detection level of the analytical methods used in the analysis.

**Table 2:** Traditional wet chemical composition for eucalyptus plants with lignin pathway modifications

Sample	% Ash	% Water extractives	% Ethanol extractives	% Lignin	% Glucose	% Xylose	% Galactose	% Arabinose	% Acetyl	% Mass closure
Control a	0.5	4.4	0.9	29.3	39.4	13.0	1.7	0.5	3.9	93.5
Control b	0.7	3.9	0.9	30.0	40.9	13.1	1.5	0.6	3.9	95.3
Control avg	0.6	4.2	0.9	29.6	40.2	13.0	1.6	0.5	3.9	94.4
C3′H 105	0.7	7.3	1.2	22.3	38.8	16.0	1.4	0.0	4.7	92.4
C3′H 106a	0.5	5.7	1.0	21.3	41.9	16.3	1.8	0.6	4.9	94.0
C3′H 106b	0.5	6.7	1.2	21.4	41.0	16.1	1.3	0.6	4.9	93.7
C3′H 113a	0.5	7.0	1.1	21.5	39.5	17.1	1.3	0.3	5.1	93.3
C3′H 113b	0.5	6.9	1.3	22.1	38.5	17.3	1.3	0.7	4.9	93.5
C3′H 114	0.9	4.6	1.7	21.6	38.5	18.4	1.3	3.1	4.9	95.1
C3′H avg	0.6	6.4*	1.3	21.7*	39.7	16.9*	1.4	0.9	4.9*	93.7
C4H 73a	0.9	3.7	0.8	17.8	46.3	16.6	2.5	0.3	4.7	93.6
C4H 73b	0.9	5.8	0.9	18.9	42.3	17.3	1.7	0.3	4.9	92.8
C4H 91	0.8	3.8	0.9	20.9	43.5	16.6	1.8	2.5	4.2	95.0
C4H 92	0.8	3.8	1.0	19.5	44.0	17.7	1.4	2.7	4.5	95.4
C4H 93	0.7	4.7	0.8	18.5	42.7	18.5	1.9	2.6	4.7	95.2
C4H 96	0.7	5.8	0.8	17.5	41.3	19.0	1.4	0.1	5.2	91.9
C4H 98	0.8	3.9	1.0	20.1	43.5	17.5	1.7	2.2	4.3	95.0
C4H 99a	0.8	4.4	1.3	18.5	41.6	18.3	1.6	0.4	4.9	91.9
C4H 99b	0.9	5.3	1.0	18.4	42.0	17.6	1.6	0.3	4.7	91.9
C4H avg	0.8*	4.6	0.9	18.9*	43.0	17.7*	1.7	1.3	4.7*	93.6

* Indicates significant difference from the control average at the *p* value <0.05 level

Thioacidolysis and PyMBMS were used to characterize changes in lignin structure and to measure the relative amounts of subunits in the lignin polymer. Thioacidolysis determined average S/G ratios of 1.5, 1.5, and 2.0 for the *C3′H* reduced, *C4H* reduced, and control samples, respectively (Table 3). Thioacidolysis measured a significant increase in the H monomers released in the *C3′H* down-regulated lines, indicating that a larger fraction of lignin is the 4-coumaryl alcohol (H) subunit. The increase in H monomers is consistent with impeding the conversion of *p*-coumaroyl quinate/shikimate to caffeoyl quinate/shikimate by down-regulation of the *C3′H* gene, reducing the formation of the monolignols necessary for G lignin and S lignin, while increasing the monolignols necessary for H lignin (see Bonawitz and Chapple [20] for a schematic of the lignin biosynthetic pathway). These results follow the same trend that was previously observed in alfalfa [27], where control and *C4H* down-regulated plants displayed similar H lignin levels of 3–4 % and the *C3′H* down-regulated lines displayed 48 % H lignin. PyMBMS confirmed that there was a reduction in the ratio of the syringyl to guaiacyl monomers from 2.1 in the control samples to 1.2 in both the *C3′H* and *C4H* RNAi lines (Table 3). PyMBMS did not detect an increase in molecular fragments that are associated with H lignin in the *C3′H* RNAi lines. It was determined that the non-pyrolyzed char content was highly correlated to the amount of H lignin as determined by thioacidolysis, indicating that the H lignin fraction was not fragmenting to smaller molecules during pyrolysis and, therefore, was not detected (data not shown). Previous ^13^C nuclear magnetic resonance (^13^C-NMR) analyses of lignin isolated from *C3′H* down-regulated lines in alfalfa have determined that there is a greater prevalence of C–C bonds in H lignin that would remain intact at the pyrolysis temperatures used in this study [31].

**Table 3:** Thioacidolysis results compared with lignin and S/G estimates using pyrolysis molecular beam mass spectrometry (PyMBMS)

Sample	Thioacidolysis				PyMBMS	
	% S	% G	% H	S/G Ratio	S/G Ratio	Lignin
Control a	66.0	32.6	1.5	2.0	2.1	30.0
Control b	65.8	33.7	0.5	1.9	2.1	30.9
Control avg	65.9	33.2	1.0	2.0	2.1	30.4
C3′H 105	51.7	34.0	14.2	1.5	1.4	22.9
C3′H 106a	48.3	34.2	17.6	1.4	1.2	20.5
C3′H 106b	46.9	34.2	19.0	1.4	1.1	21.2
C3′H 113a	49.1	32.4	18.5	1.5	1.2	21.5
C3′H 113b	48.8	31.2	19.9	1.6	1.2	20.6
C3′H 114	46.1	33.5	20.4	1.4	1.1	19.6
C3′H avg	48.5*	33.2	18.3*	1.5*	1.2*	21.0*
C4H 73a	58.6	38.2	3.1	1.5	1.2	21.9
C4H 73b	59.0	40.2	0.9	1.5	1.2	21.9
C4H 91	57.7	41.4	0.9	1.4	1.3	23.1
C4H 92	58.2	41.2	0.7	1.4	1.3	22.5
C4H 93	59.2	39.7	1.1	1.5	1.2	21.1
C4H 96	58.4	40.8	0.8	1.4	1.2	21.8
C4H 98	61.5	37.6	0.9	1.6	1.3	22.9
C4H 99a	60.4	38.5	1.2	1.6	1.1	19.0
C4H 99b	58.0	40.9	1.0	1.4	1.1	21.2
C4H avg	59.0*	39.8*	1.2	1.5*	1.2*	21.7*

* Indicates significant difference from the control average at the *p* value <0.05 level

### Reduced recalcitrance

Wood samples were analyzed using a high-throughput pretreatment and enzymatic saccharification process to determine if the *C3′H* and *C4H* gene expression modifications lowered recalcitrance [32, 33]. Reduced recalcitrance is defined as an increase in glucose and xylose release after enzymatic saccharification compared to the control samples under a defined pretreatment condition. The cell wall chemistry data of the sugar content in the biomass allowed the sugar release data to be converted to a percent release for both non-pretreated and pretreated samples. For samples not subjected to pretreatment, the *C4H* reduced lines released an average 21 % of the total sugar, which was a significant improvement over the control samples that displayed an average of only 2 % of the total sugars released, while the *C3′H* reduced lines averaged a 16 % release (Table 4). Chen and Dixon [7] also found decreased recalcitrance in alfalfa lines with down-regulated *C3′H* expression, with enzymatic hydrolysis efficiency approximately twice that of the controls for the *C3′H* antisense lines and 1.5 times the control for the *C4H* antisense lines. In the present study on eucalyptus, hot water pretreated samples displayed an average percent total sugar release for the control of 80 %, while the *C3′H* down-regulated lines released 94 % of the total sugars and the C4H lines 97 %. By providing a near 100 % release upon pretreatment, these lines have high potential for bioconversion. Plants requiring decreased or no pretreatment are of interest for the biofuel industry because pretreatment is often one of the most costly aspects of fuel production due to chemical costs, sugar degradation products that can inhibit fermentations, and capital costs [6].

**Table 4:** Percentage of sugar released by enzymatic saccharification

Sample	No pretreatment			Hydrothermal pretreatment		
	% Glucose	% Xylose	% Total	% Glucose	% Xylose	% Total
Control a	3	1	3	72	97	78
Control b	1	2	1	73	108	82
Control avg	2	1	2	73	102	80
C3′H 105	11	9	11	89	111	95
C3′H 106a	17	11	15	86	106	92
C3′H 106b	16	13	15	86	110	92
C3′H 113a	16	11	14	87	109	93
C3′H 113b	16	13	15	90	110	96
C3′H 114	29	25	28	82	97	87
C3′H avg	18*	14*	16*	87*	107	93*
C4H 73a	30	21	28	82	103	88
C4H 73b	18	14	17	94	113	99
C4H 91	12	9	11	88	107	93
C4H 92	15	11	14	89	103	93
C4H 93	26	17	23	94	105	97
C4H 96	23	16	21	96	103	98
C4H 98	22	12	19	84	96	87
C4H 99a	30	22	28	98	111	102
C4H 99b	28	23	26	93	109	98
C4H avg	23*	16*	21*	91*	106	95*

* Indicates significant difference from the control average at the *p* value <0.05 level

However, genetically modified plants with strong lignin reduction typically result in reduced growth, which decreases per-acre crop yields [20], and the mechanisms behind this are still being investigated [34]. While the *C3′H* down-regulated lines exhibited severely reduced growth (average height 2.0 m compared to 6.0 m for the control), the *C4H* down-regulated lines showed a milder effect (average height 3.4 m); and there were no obvious pest problems observed for the reduced-lignin lines. It may be possible to mitigate the negative growth effect through cultural treatments such as irrigation or coppicing. In addition, more refined lignin modification constructs that protect the integrity of vessel walls could to be other pathways toward the protection of yield. A promoter with expression restricted to fibers might be used in down-regulation constructs. Another alternative might be introduction of a *C4H* gene under control of a vessel-specific promoter in combination with down-regulation or mutation of the more generally expressed endogenous *C4H* gene [35]. Despite the slower growth, the *C4H* down-regulated wood responded well to a mild hot water pretreatment; therefore, the reduced lignin trees may still provide economic benefit. Hot water pretreatment has the potential to significantly lower the cost associated with biomass pretreatment due to a reduction in chemical usage, lower amounts of sugar degradation products that can inhibit fermentation, and capital costs compared to other pretreatment technologies, such as dilute acid, alkali, and steam explosion [32, 33].

**Fig. 2:**
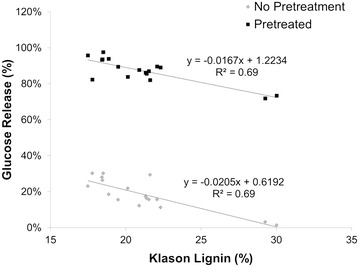
Glucose release versus Klason lignin content for samples that were not subjected to pretreatment (*gray diamonds*) and pretreated samples (*black squares*)

The results of this study determined that lignin content has a large impact on lowering recalcitrance, as shown by the correlation between Klason lignin content and the percentage of glucose released after pretreatment and enzymatic hydrolysis (Fig. 2). The down-regulation of the *C4H* gene produced trees that had an average Klason lignin content of 17.8 % and released 91 % of the available glucose compared to 73 % for the control under identical pretreatment conditions. The low lignin lines also released significantly more sugar without pretreatment, although pretreatment is still necessary to release enough sugar to make these transgenic lines candidates for biofuel production. These results agree well with published data on alfalfa where lignin levels were observed to correlate with sugar release [7]. Reducing recalcitrance by reducing lignin content was also reported by Fu et al. [8] via an ethanol assay with *COMT* down-regulated switchgrass, where a 12–15 % reduction in lignin content resulted in an increased ethanol yield of up to 38 % over the control.

With the strong correlation between lignin content and glucose release, the question is raised as to whether the reduction in recalcitrance is also associated with the lignin structure. The wood samples produced by down-regulation of either the *C4H* or *C3′H* genes have similar S/G ratios, 1.2 as determined by PyMBMS and 1.4 from thioacidolysis. However, the *C4H* RNAi lines released greater amounts of glucose than the *C3′H* RNAi lines utilizing no pretreatment and hot water pretreatment. In addition, these sugar release results indicate that H lignin content is not, in and of itself, a primary factor in recalcitrance since the *C3′H* down-regulated lines, containing 18.3 % H lignin (Table 3), were more recalcitrant than the *C4H* down-regulated lines with only 1.2 % H lignin. Therefore, in this study, lowering lignin content appears to have had a much larger effect on reducing recalcitrance than changing the H lignin subunit ratio. Our results are consistent with the findings of Chen and Dixon [7], finding no real correlation between S/G ratio and sugar release for antisense C3′H and antisense shikimate hydroxycinnamoyl transferase (HCT) low lignin transgenic lines in alfalfa. However, these eucalyptus results are not in agreement with data from natural poplar variants that were found to be less dependent on lignin content and more dependent on the S/G ratio of the wood samples [32, 36].

## Conclusions

Down-regulating *C3′H* and *C4H* genes in the lignin biosynthetic pathway results in eucalyptus trees with lower lignin content and altered S/G/H ratios. Thioacidolysis results indicate that the *C3′H* RNAi lines contain an average 18.3 % H lignin, while the *C4H* RNAi lines contain only 1.2 %, similar to the control samples at 1 % H lignin. Down-regulation of each gene led to lines with significantly reduced recalcitrance, releasing a greater amount of glucose with lower severity pretreatments. The *C4H* down-regulated lines had particularly high sugar release, with more than half the lines achieving 90 % glucose release using hot water pretreatment, with two lines having glucose release of more than 95 %. This study identified lowering lignin content rather than altering the S/G/H ratios had the largest impact on reducing recalcitrance of the transgenic lines. The less recalcitrant nature of the *C3′H* and *C4H* RNAi lines make these plants excellent candidates for biofuel feedstocks.

## Methods

### Plant materials

To test the effect of different target genes on reducing lignin in hybrid eucalyptus, *E. urophylla* × *E. grandis* was transformed with two plasmids designed to reduce the expression of genes in the lignin biosynthetic pathway [37]. Gene sequence information for the *E. grandis* genome [38] can be obtained at the Phytozome website [39, 40]. *EgrC4H1* [Phytozome:Eucgr.J01844] and *EgrC3′H3* [Phytozome: Eucgr.A02190] cDNAs were isolated from xylem libraries derived from *E. grandis*. For each gene, a 600-bp fragment from the 3′ portion of the cDNA was cloned as an inverted repeat, driven by the promoter from a *E. grandis* arabinogalactan protein gene [Phytozome:Eucgr.B02846]. The xylem-preferred promoter comprised 2340 base pairs upstream of the predicted initiation codon for this gene [37]. The plasmid identities were pARB669 for *EgrC3′H3* and pARB670 for *EgrC4H1*. The plasmids were introduced into an in vitro clonally propagated line of *E. urophylla* × *E. grandis* via *Agrobacterium*-mediated transformation of young leaves similar to that described by Tournier et al. [41]. Fifteen independent events were generated for pARB669 and 18 events were produced for pARB670. The nontransformed line was used as the control. The plants were grown for 3 months as containerized cuttings in the greenhouse, acclimatized for 1 month outdoors, and then transferred into the field in Glades County, Florida, for an additional 20 months before destructive sampling. Stems were debarked, air dried, and milled with a Wiley mill to pass through a 20-mesh screen.

### Gene expression

Gene expression was measured using real-time qPCR. For these analyses, additional plants of a subset of the lines were established in a greenhouse and grown for 6 months. Three lines transformed with a selectable marker were used as controls. Lignifying lateral branches approximately 2 mm in diameter were collected without removing pith or epidermis. Samples were frozen in liquid N_2_ and stored at −80 °C until RNA extraction.

Total RNA was extracted from 100 mg samples of greenhouse-grown stem material, which had been ground to a powder with mortar, pestle, and liquid nitrogen, using the RNeasy Plant Mini Kit (QIAGEN). Potentially contaminating genomic DNA was removed from the RNA by treatment with the TURBO DNA-free Kit (Ambion). cDNA was synthesized using the Affinity Script qPCR cDNA Synthesis Kit (Stratagene), using between 20 and 100 ng of total RNA per sample. All kits were used according to their respective manufacturer’s instructions.

Quantitative PCR was performed in a 96-well format using an Agilent Technologies/Stratagene Mx3000P with an elongation factor 1α gene [Phytozome:Eucgr.G02807] used as the reference. The qPCR primers and probes were designed using Primer3 [42]. Primer oligonucleotides were synthesized by Invitrogen, and probes were synthesized by Biosearch Technologies. The primer oligonucleotides were C4H-J01844-L1 (5′-ATCTGCAAGGAGGTCAAGGA-3′), C4H-J01844-R1 (5′-CGTTGATGTTCTCGACGATG-3′), C3H-A02190-L1 (5′-GCACCAACCCTGATAATTCG-3′), C3H-A02190-R1 (5′-GACACGATCGCCTTGAACTC-3′), EFA-G02807-L2 (5′-GGGCCCCACCCTCCTCGACGCT-3′), and EFA-G02807-R1 (5′-GCCGTTGCCAATCTGCCCGGGGT-3′). Probes for the lignin genes were C4H-J01844-P1 (5′-ACTTCGTCGACGAGAGGAAA-3′) and C3H-A02190-P1 (5′-TGGTGAAGAAGTACCTGGGG-3′), each labeled with 6-carboxyfluorescein. The probe for the reference gene, EFA-G02807-P2 (5′-AGGCTCTCCAGGAGGCCCTCCCT-3′), was labeled with hexachlorofluorescein.

Each 25 μL reaction contained 3 pmol of the appropriate probe oligonucleotide and 5 pmol of each corresponding primer oligonucleotide, plus 12.5 μL of Maxima Probe/ROX qPCR Master Mix (2×) (Thermo Fisher Scientific #K0233) and cDNA equivalent to 1–5 ng of total RNA. Three plants were analyzed from each line. A single RNA purification and cDNA synthesis was done for each biological replicate and all qPCR reactions were run in duplicate. Changes in expression of the genes of interest in the RNAi lines relative to their expression in the transformed controls were calculated by the ddCt method [41], using the average of the dCt of each gene in the control lines as the baseline for that gene.

### Removal of extractives

Dried biomass samples were knife milled to 60-mesh and loaded into tea bags and sealed for extraction. The samples were extracted with a 1:1 water and ethanol mixture using a soxhlet and allowed to air dry in a hood for 24 h.

### Starch content

Starch content was measured on all samples to ensure starch did not artificially contribute to the sugar release values. The starch assay was adapted from a standard commercial starch test from Megazyme International. After loading the biomass into the wells of a 96-well Hastelloy plate, each of the samples was wetted with 20 µL of 80 % ethanol, and 300 µL of the amylase solution was added. The plates were sealed with a polypropylene adhesive seal (Titer-Tops, Diversified Biotech) augmented with custom magnetic sealing lids. The sealed plates were incubated overnight at 55 °C and 125 rpm in a HiGro microtiter plate incubator. Glucose analysis after amylase digestion was conducted using the glucose oxidase assay described in Selig et al. [32].

### Recalcitrance measurements

Recalcitrance was determined using a high-throughput system developed at the National Renewable Energy Laboratory (NREL) for the BioEnergy Science Center as described in Selig et al. [32] and Decker et al. [33]. Briefly, control and experimental biomass samples were knife milled to pass a 20-mesh screen and then dispensed into a custom-machined, 96-well Hastelloy plates in 5.0 ± 0.3 mg/well aliquots using the Symyx robotic system. Triplicate samples, as well as standards and controls, were distributed across the plates. Water was added to each well (250 μL), and the loaded reactor plate was sealed using silicone adhesive Teflon tape and sandwiched between two empty plates. Glass reinforced Teflon gaskets were placed between adjacent plates, and then the stack was clamped tightly using a custom clamping system. The clamped stack was then loaded into a Parr reactor electrically preheated to 180 °C. Steam was introduced and the reactor assembly was held at 180 °C (as determined by a thermocouple in the reactor vessel) for 17.5 min. Steam was then vented and cooling water was allowed to flow into the reactor. These pretreatment conditions were chosen to be suboptimal to enhance differences due to changes in the biomass recalcitrance. The suboptimal conditions were chosen to release 70 % of the glucose of the standard *Populus trichocarpa* × *P. deltoides* F1 hybrid, clone 53–239 sample harvested near Oak Ridge, Tennessee.

Enzymatic saccharification of the samples with no pretreatment and pretreated solids including the hydrolysate was carried out in the 96-well reactor plates. All the saccharification samples were loaded with an excess (70 mg/g initial biomass) of a commercial enzyme preparation CTec2 (Novozyme 188, Novozymes A/S, Bagsværd, Denmark), buffered with 1 M citrate to maintain a pH of 5.0, and incubated at 50 °C for 72 h. Analysis of glucose and xylose released into the hydrolysate from the enzymatic saccharification process was performed using glucose oxidase/peroxidase and xylose dehydrogenase assays (Megazyme International, Wicklow, Ireland).

### Cell wall compositional analysis

The biomass composition (glucan, xylan, galactan, arabinan, lignin, and ash) was determined by following the NREL Laboratory Analytical Procedure (LAP): Determination of Structural Carbohydrates and Lignin in Biomass (NREL/TP-510-42618). NREL LAPs are available online [43].

### Lignin characterization

A commercially available molecular beam mass spectrometer modified for biomass analysis was used for pyrolysis vapor analysis [44–46]. Approximately 4 mg of air-dried, 20-mesh biomass was pyrolyzed at 500 °C. Mass spectral data from *m/z* 30–450 were acquired using 17 eV electron impact ionization. S/G ratios were estimated by summing the syringyl peaks 154, 167, 168, 182, 194, 208, and 210 and dividing by the sum of guaiacyl peaks 124, 137, 138, 150, 164, and 178 [44]. Several lignin peaks were omitted in the syringyl or guaiacyl summations due to individual peaks having associations with both S and G precursors [44]. Thioacidolysis for analyzing S, G, and H monomer content was performed according to the procedure described in Foster et al. [47]. Thioacidolysis is used to determine the H monomer contribution due to the inability of the pyrolysis method to efficiently detect this monomer.

## Abbreviations

^13^C-NMR: carbon-13 nuclear magnetic resonance
C3′H: *p*-coumaroyl quinate/shikimate 3′-hydroxylase or *p*-coumarate 3-hydroxylase
C4H: cinnamate 4-hydroxylaseCOMT—caffeic acid 3-*O*-methyltransferase
dCt: delta threshold value
ddCt: delta–delta threshold value
EC: Enzyme Commission
G: coniferyl alcohol
H: 4-coumaryl alcohol
HCT: shikimate hydroxycinnamoyl transferase
LAP: Laboratory Analytical Procedure
NREL: National Renewable Energy Laboratory
PyMBMS: pyrolysis molecular beam mass spectrometry
qPCR: quantitative polymerase chain reaction
RNAi: RNA interference
S: sinapyl alcohol
